# Research on radiotherapy related genes and prognostic target identification of rectal cancer based on multi-omics

**DOI:** 10.1186/s12967-023-04753-9

**Published:** 2023-11-27

**Authors:** Yi Liu, Yanguang Yang, Feng Ni, Guomei Tai, Cenming Yu, Xiaohui Jiang, Ding Wang

**Affiliations:** 1 Department of Radiotherapy, Affiliated Tumor Hospital of Nantong University, Nantong Tumor Hospital, Nantong, Jiangsu China; 2 Department of Gastrointestinal Surgery, Affiliated Tumor Hospital of Nantong University, Nantong Tumor Hospital, Nantong, Jiangsu China

**Keywords:** Rectal cancer, Differentially expressed genes, Radiosensitivity, Multi-omics, Prognosis

## Abstract

**Background:**

Radiosensitivity of rectal cancer is related to the radiotherapy efficacy and prognosis of patients with rectal cancer, and the genes and molecular mechanisms related to radiosensitivity of rectal cancer have not been clarified. We explored the radiosensitivity related genes of rectal cancer at a multi omics level.

**Methods:**

mRNA expression data and rectum adenocarcinoma (READ) data were obtained from the Cancer Genome Atlas (TCGA) and the Gene Expression Omnibus Database (GEO) (GSE150082, GSE60331, GSE46862, GSE46862). Differentially expressed genes between radiotherapy sensitive group and radiotherapy insensitive group were screened. GO analysis and KEGG pathway analysis were performed for differentially expressed genes. Among the differentially expressed genes, five core genes associated with rectal cancer prognosis were selected using random survival forest analysis. For these five core genes, drug sensitivity analysis, immune cell infiltration analysis, TISIDB database immune gene correlation analysis, GSEA enrichment analysis, construction of Nomogram prediction model, transcriptional regulatory network analysis, and qRT-PCR validation was performed on human rectal adenocarcinoma tissue.

**Results:**

We found that 600 up-regulated genes and 553 down-regulated genes were significantly different between radiotherapy sensitive group and radiotherapy insensitive group in rectal cancer. Five key genes, TOP2A, MATR3, APOL6, JOSD1, and HOXC6, were finally screened by random survival forest analysis. These five key genes were associated with different immune cell infiltration, immune-related genes, and chemosensitivity. A comprehensive transcriptional regulatory network was constructed based on these five core genes. qRT-PCR revealed that MATR3 expression was different in rectal cancer tissues and adjacent non-cancerous tissues, while APOL6, HOXC6, JOSD1, and TOP2A expression was not different.

**Conclusion:**

Five radiosensitivity-related genes related to the prognosis of rectal cancer: TOP2A, MATR3, APOL6, JOSD1, HOXC6, are involved in multiple processes such as immune cell infiltration, immune-related genes, chemosensitivity, signaling pathways and transcriptional regulatory networks and may be potential biomarkers for radiotherapy of rectal cancer.

## Introduction

Rectal cancer is a common malignant tumor of the digestive tract. By the end of 2022, according to the latest epidemiological survey, rectal cancer was the third most common tumor worldwide. For patients with stage I rectal cancer, if the tumor is close to the anus, local tumor resection + postoperative radiotherapy can be given, and the same efficacy as radical surgery can be obtained while preserving the anus. For patients with stage II-III rectal cancer, preoperative concurrent chemoradiotherapy and postoperative concurrent chemoradiotherapy reduced locoregional recurrence rates and significantly improved long-term survival compared with surgery alone. Compared with postoperative concurrent chemoradiotherapy, preoperative concurrent chemoradiotherapy achieves similar long-term survival, and on this basis further reduces the locoregional recurrence rate and the incidence of adverse reactions, and improves the sphincter preservation rate, and preoperative concurrent chemoradiotherapy becomes the standard method for stage II–III surgically respectable rectal cancer [1]. For patients with locally advanced inoperable rectal cancer, preoperative concurrent chemoradiotherapy can make some patients obtain the chance of surgery, while for patients who are still inoperable, palliative reduction can be performed. After preoperative concurrent chemoradiotherapy for locally advanced rectal cancer, radical surgical pathology confirmed a pCR rate between 12 and 20% [2]. Patients who achieved pCR had improved overall and disease-free survival and reduced local recurrence rates compared with those who did not achieve pCR. At present, there is no elucidation on the related genes predicting the sensitivity of radiotherapy for rectal cancer and the pCR status after preoperative concurrent chemoradiotherapy for rectal cancer in clinical and basic research, so this paper intends to explore the related genes of radiotherapy sensitivity for rectal cancer from the multi-omics direction.

Tumor radiosensitivity is controlled by multiple factors such as DNA damage repair, regulation of cell cycle checkpoints, regulation of signal transduction pathways, and tumor microenvironment. The target of ionizing radiation is DNA. DNA double-strand breaks (DSBs) are lethal lesions, and their repair is mainly achieved by non-homologous end joining (NHEJ) and homologous recombination (HR). HR occurs mainly in the S and G_2_ phases of the cell cycle, and NHEJ is mainly achieved by DNA-PKcs, KU70, and KU80, and inhibition of important targets in DSB repair such as ATM or DNA-PK significantly improves radiosensitivity [3]. Cell cycle checkpoint inhibitors increase radiosensitivity in tumor cells. The main pathways associated with radiosensitivity are PI3k–AkT, NF-κB, MAPK and TGFβ, the first three of which are associated with cell survival, and TGFβ may affect radioresistance by controlling ATM activation. Hypoxic status, hypoxia-inducible factor (HIF), tumor angiogenesis are also associated with radiotherapy. Other studies such as cancer stem cells, microRNAs and radiosensitivity have also been reported, but most biomarkers lack sensitivity and specificity.

In recent years, significant progress has been made in cancer immunotherapy, but in clinical practice, immunotherapy alone has brought benefits to only a small proportion of cancer patients, while radiation therapy can release tumor-specific antigens from tumor cells, activate dendritic cells with antigen presentation around the antigen, drain activated dendritic cells to lymph nodes and T cells to produce systemic anti-tumor immune responses, and at the same time, the induced use of immunotherapy can also change the tumor microenvironment, promote tumor angiogenesis, improve hypoxia, and produce a synergistic effect with radiotherapy, and radiotherapy combined with immunotherapy can transform immune-cold tumors into immune-hot tumors and enhance anti-tumor immune effects [4]. However, the specific role of radiotherapy combined with immunization in the treatment of rectal cancer has not been elucidated. Nanomaterials have been extensively studied in cancer therapy as vectors that may improve drug delivery. Such vectors not only bring numerous advantages such as stability, biocompatibility, and cellular uptake but have also been shown to overcome some cancer-related resistances [5–7].

In summary, the mechanisms involved in chemoradiotherapy and immunotherapy sensitivity in rectal cancer remain elusive, therefore, we comprehensively explore the gene differences between radiotherapy sensitive group and radiotherapy insensitive group in rectal cancer, find five genes closely related to prognosis in differential genes, and then comprehensively explain the distribution of cell signaling pathways, immune infiltration, correlation of immune genes, sensitivity of chemotherapeutic drugs, and transcription factor regulation of these five genes in order to find appropriate biomarkers to guide clinical practice.

## Method

### Data acquisition

The TCGA database (READ dataset) (https://portal.gdc.cancer.gov/), as the largest current cancer gene information database, stores data including gene expression data, miRNA expression data, copy number variation, DNA methylation, and SNPs. We first downloaded processed raw mRNA expression data for READ, including normal (n = 9), tumor (n = 152) groups. The Series Matrix File data file for GSE150082 [8] was downloaded from the NCBI GEO (https://www.ncbi.nlm.nih.gov/geo/) public database with the annotation platform GPL13497, and 39 READ patient data with complete expression profiles and clinical information were extracted. Download Series Matrix File data file of GSE60331 [9] with annotation platform GPL15207, and extract 31 READ patient data with complete expression profile and clinical information. Download Series Matrix File data files of GSE46862 with annotation platform GPL6244, and extract 69 READ patient data with complete expression profiles and clinical information. Download GSE35452’s Series Matrix File data file with annotation platform GPL570, and extract 46 READ patient data with complete expression profiles and clinical information.

### Random survival forest [10] screening critical genes

Feature selection was performed using the randomForestSRC software package. We also ranked the importance of prognostically relevant genes using a random survival forest algorithm (nrep = 1000, which suggests that the number of iterations in Monte Carlo simulations is 1000). We identified genes with relative importance > 0. 3 as final marker genes.

### GO and KEGG function analysis

clusterProfiler [11] was used to functionally annotate differential genes to comprehensively investigate the functional relevance of these differential genes. Gene Ontology (GO) and Kyoto Encyclopedia of Genes and Genomes (KEGG) were used to assess the associated functional categories. GO and KEGG enriched pathways with both p-values and q-values less than 0.05 were considered as significant categories.

### Drug sensitivity analysis

Based on the largest pharmacogenomics database (GDSC Cancer Drug Sensitivity Genomics Database, https://www.cancerrxgene.org/), we used the R software package “pRRophetic” to predict chemosensitivity for each tumor sample; methods of regression yielded IC50 estimates for each specific chemotherapeutic agent treatment, and 10 cross-validation test regression and prediction accuracy with the GDSC training set. Default values were chosen for all parameters, including removing “combat” [12] from batch effects and taking the average of duplicate gene expression.

### Immune cell infiltrate analysis

RNA -seq data from READ patients in different subgroups were analyzed using the CIBERSORT [13] algorithm to infer the relative proportions of 22 immune infiltrating cells, and spearman correlation analysis was performed for gene expression as well as immune cell content, *P* < 0.05 was considered statistically different.

### GSEA enrichment analysis

Gene set enrichment analysis (GSEA; http://www.broadinstitute.org/gsea), to identify genes differentially expressed between patients in the high and low expression groups. Gene sets were filtered using maximum and minimum gene set sizes of 500 and 15 genes, respectively. A 100 permutation was performed to obtain a rich gene set based on a p-value < 0.05 and a false discovery rate (FDR) value of 0.25.

### TISIDB database immunogene correlation analysis

TISIDB (http://cis.hku.hk/TISIDB/index.php) is an online website for tumor and immune system interactions, which integrates multiple heterogeneous data types that are integrated into multiple classes of information for each gene. TISIDB fuses data information from multiple databases (TCGA, UniProt, GO, DrugBank, etc.) and is a valuable resource for cancer immunological research and treatment. The immune gene set used to investigate immune system interactions in tumors was derived from the TISIDB database.

### Transcriptional regulatory network analysis

Cistrome DB (http://cistrome.org/db) is currently a relatively comprehensive database for studying ChIP-seq and DNase-seq and includes transcription factor, histone modification, and chromatin accessibility samples from 30,451 humans and 26,013 mice. In this study, we investigated the regulatory relationship of transcription factors and key genes through the Cistrome DB database, in which the genome file was set at hg38 and the transcription start site was set at 10 kb, and visualized by cytoscape.

### RNA extraction and quantitative real‑time polymerase chain reaction (qRT‑PCR)

The total RNA from 20 pairs of human rectal adenocarcinoma tissues and adjacent tissues was extracted by Trizol reagent (Thermo scientific, USA), and RNA concentration was measured. Subsequently, reverse transcription of RNA into cDNA was executed with RevertAid First Strand cDNA Synthesis Kit (Thermo scientific, USA) according to the standard instructions. Real time fluorescence quantitative PCR instrument (Analytik Jena AG, Germany) was performed for qRT-PCR to detect mRNA expression. All the primers were synthesized by Kamu Biology (Kamu, Shanghai, China). The relative expression level of mRNA was calculated by 2 − ΔΔCt method and three replicate experiments were involved. All specific primers were shown in Table 1.

**Table 1:** Primers used for real-time PCR

Gene	Primer sequence (5ʹ to 3ʹ)
APOL6	Forward: ACCAGGCGGAGAGAGAAAGT
	Reverse: TGTAGCTCCACGTCTTCACAC
MATR3	Forward: ATCAATGGAGCAAGTCACAGTC
	Reverse: TGCAACATGAATGGATCACCC
HOXC6	Forward: AGCTCCATCTCGTAACACCA
	Reverse: TCTGCCACTCTGAAACTGTTT
JOSD1	Forward: GGGATACGCTGCAAGAGATTT
	Reverse: CCATGACGTTAGTGAGGGCA
TOP2A	Forward: CTTAATACCGATCTACATGGCCA
	Reverse: CTGCTTCCACATTGCTGCTG
GAPDH	Forward: GCCGTCTAGAAAAACCTGCC
	Reverse: CCACCTGGTGCTCAGTGTAG

### Statistical analysis

All statistical analyses were performed in R language (version 3.6). All statistical tests were two-sided and* p* < 0.05 was considered statistically significant.

## Results

### To investigate the expression profile of samples sensitive to radiotherapy in the READ cohort

**Fig. 1:**
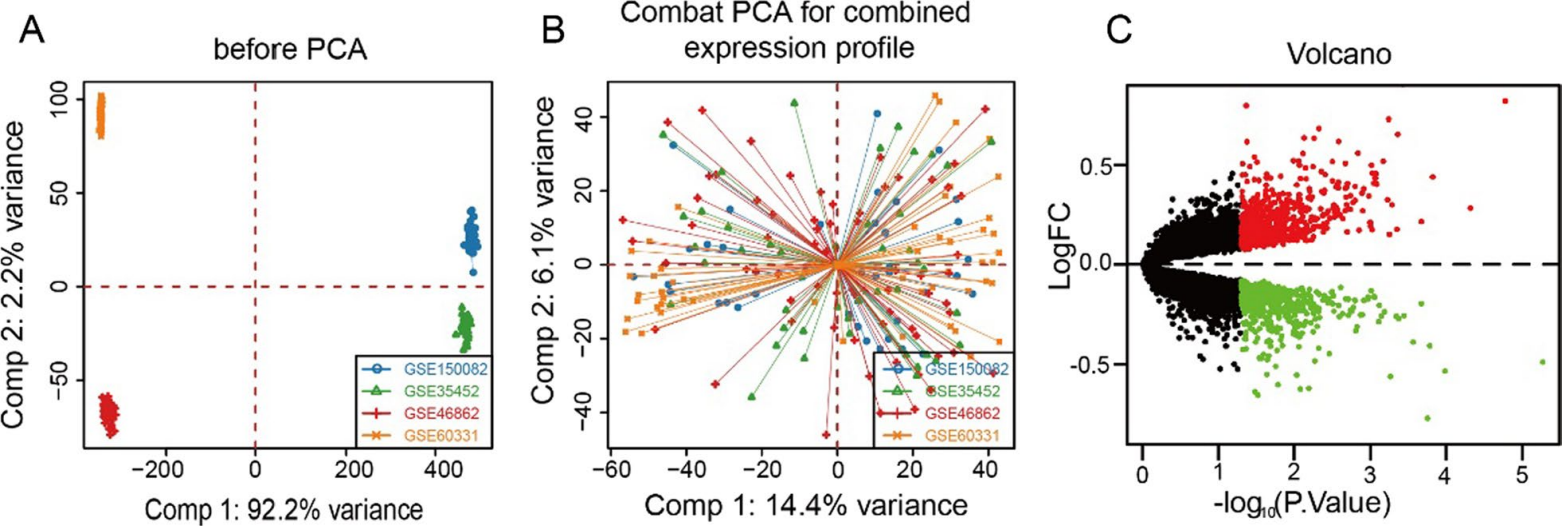
Characteristics and differences of radiosensitive genes in rectal cancer. **A**, **B** Sample expression profile. **C** Volcanic distribution plot of differential genes

We merged the four GEO datasets GSE150082, GSE60331, GSE46862, GSE35452 into an expression profile of 1 85 samples by ComBat function (radiosensitive group: 83 cases; radiosensitive group: 102 cases), and PCA plot showed that the batch effect between the merged samples disappeared (Fig. 1A, B). We found 1,153 genes with significant expression levels between radiotherapy sensitive group and radiotherapy insensitive group by limma [14] differential analysis (*p* < 0.05), containing 600 up-regulated genes and 553 down-regulated genes (Fig. 1C).

### Functional enrichment of differential gene

**Fig. 2:**
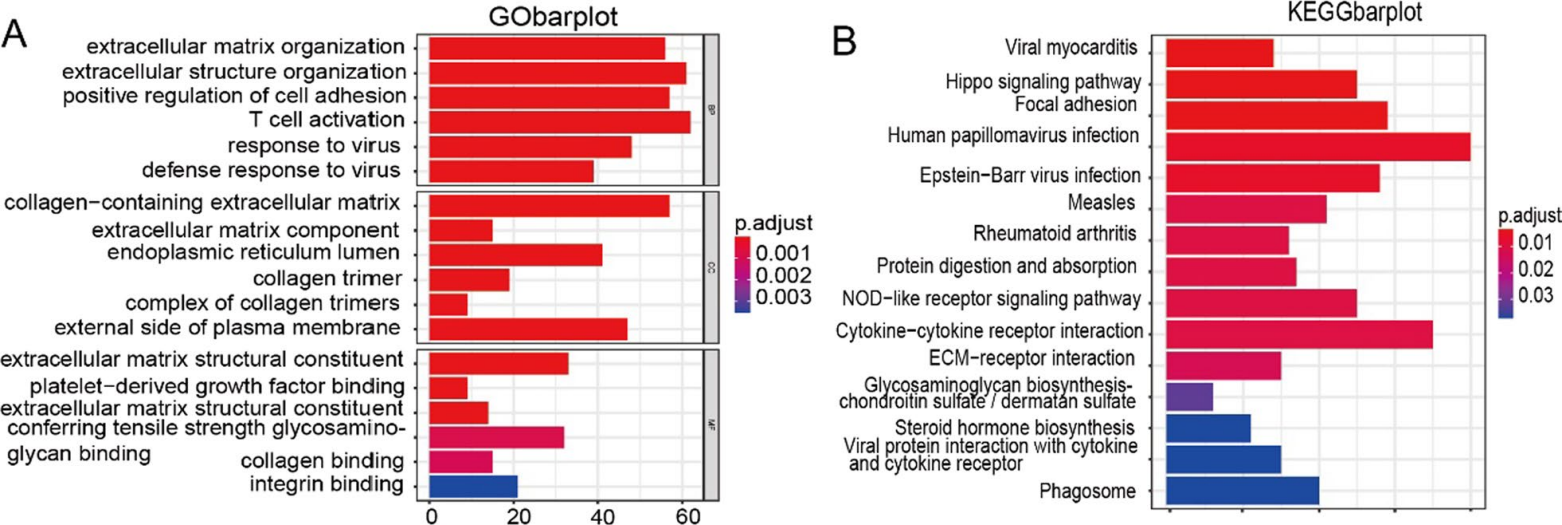
Functional enrichment of differential genes and construction of protein interaction networks. **A** Associated signaling pathways enriched in differential gene GO. **B** Associated signaling pathways enriched by differential gene KEGG

Differential gene-related signaling pathways were investigated by GO and KEGG enrichment analysis. The results showed that T cell activation, extracellular structur e organization, extracellular matrix organization and other pathways were significantly enriched in GO enrichment (Fig. 2A); Hippo signaling pathway, NOD—like receptor signaling pathway, Cytokine—like receptor cytokine interaction and other pathways were significantly enriched in KEGG enrichment (Fig. 2B).

### Random survival forest analysis of differential genes

**Fig. 3:**
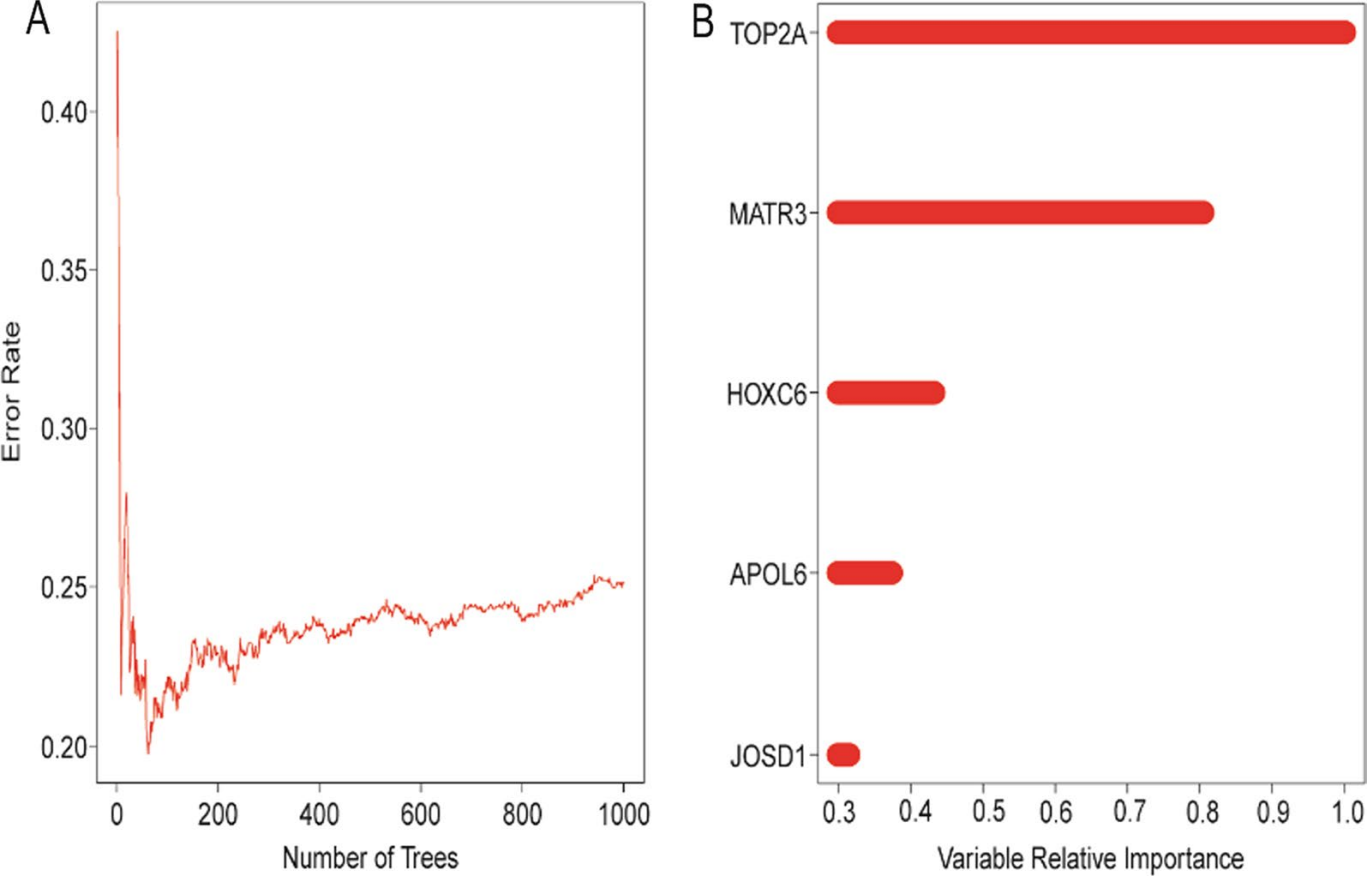
Random survival forest analysis identified 5 core genes

In order to further identify the core genes affecting the prognosis of rectal cancer among the key genes, we performed random survival forest analysis of these 1,153 genes, and we identified genes with relative importance > 0.3 as final markers, and finally screened five key genes, TOP2A, MATR3, HOXC6, APOL6, and JOSD1 (Fig. 3).

(Radiosensitive) up-regulated genes:

TOP2A (logFC: 0.22059804086761, P: 0.0327297470567203).

MATR3 (logFC: 0.184287854672041, P: 0.0108217396299998).

JOSD1 (logFC: 0.13076305951302, P: 0.0338021189456651).

APOL6 (logFC: 0.172758054291133, P: 0.0130113211634834).

(Radiosensitive) down-regulated genes:

HOXC6 (logFC: -0.287435745479151, P: 0.0430642209078996).

### Relationship between critical genes and immune infiltration

**Fig. 4:**
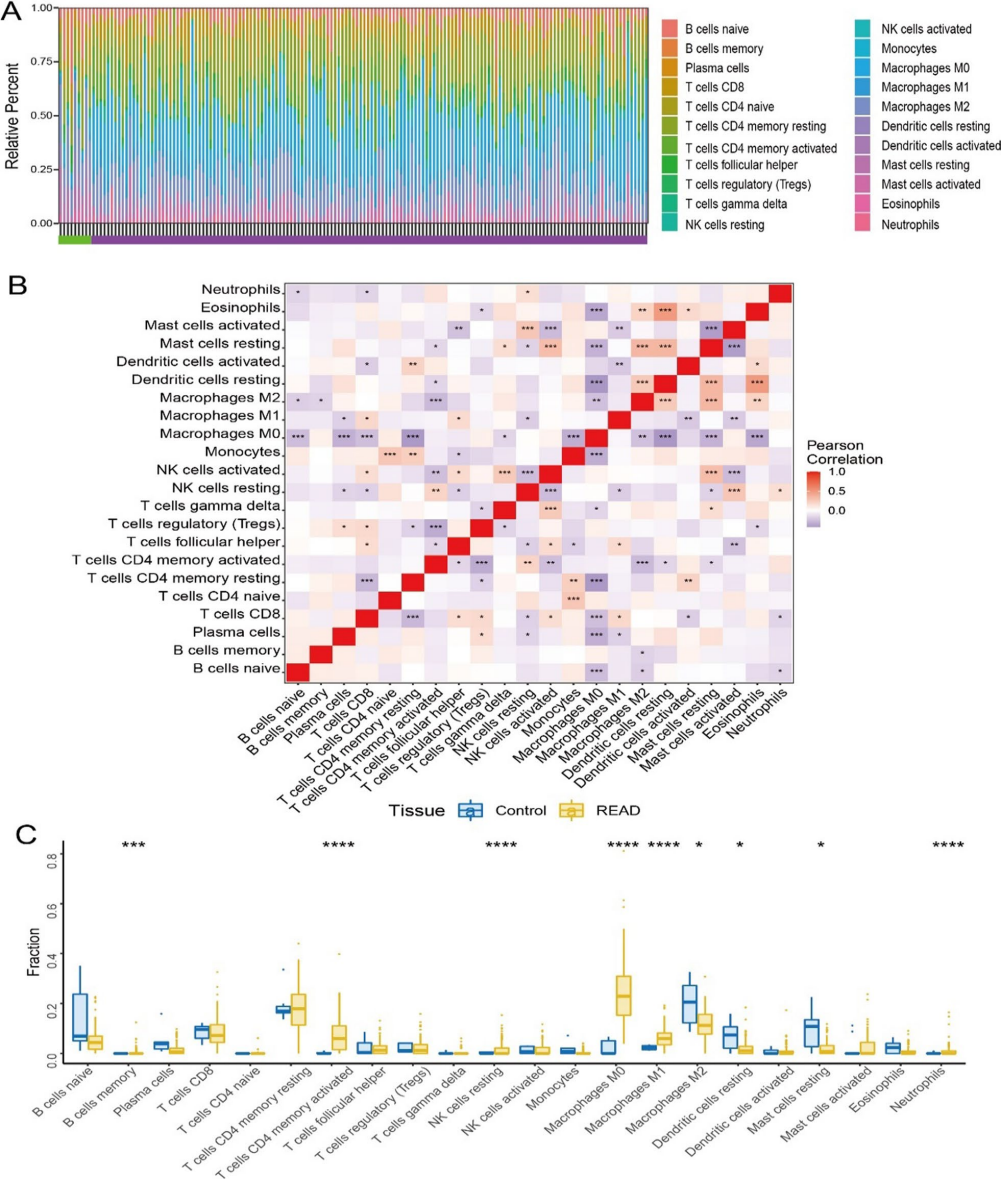
Relationship between key genes of radiotherapy sensitivity and immune infiltration in rectal cancer. **A** Percentage of immune cells. **B** Pearson correlation of immune cells.**C** Comparison of immune cell content between Read group and normal group

**Fig. 5:**
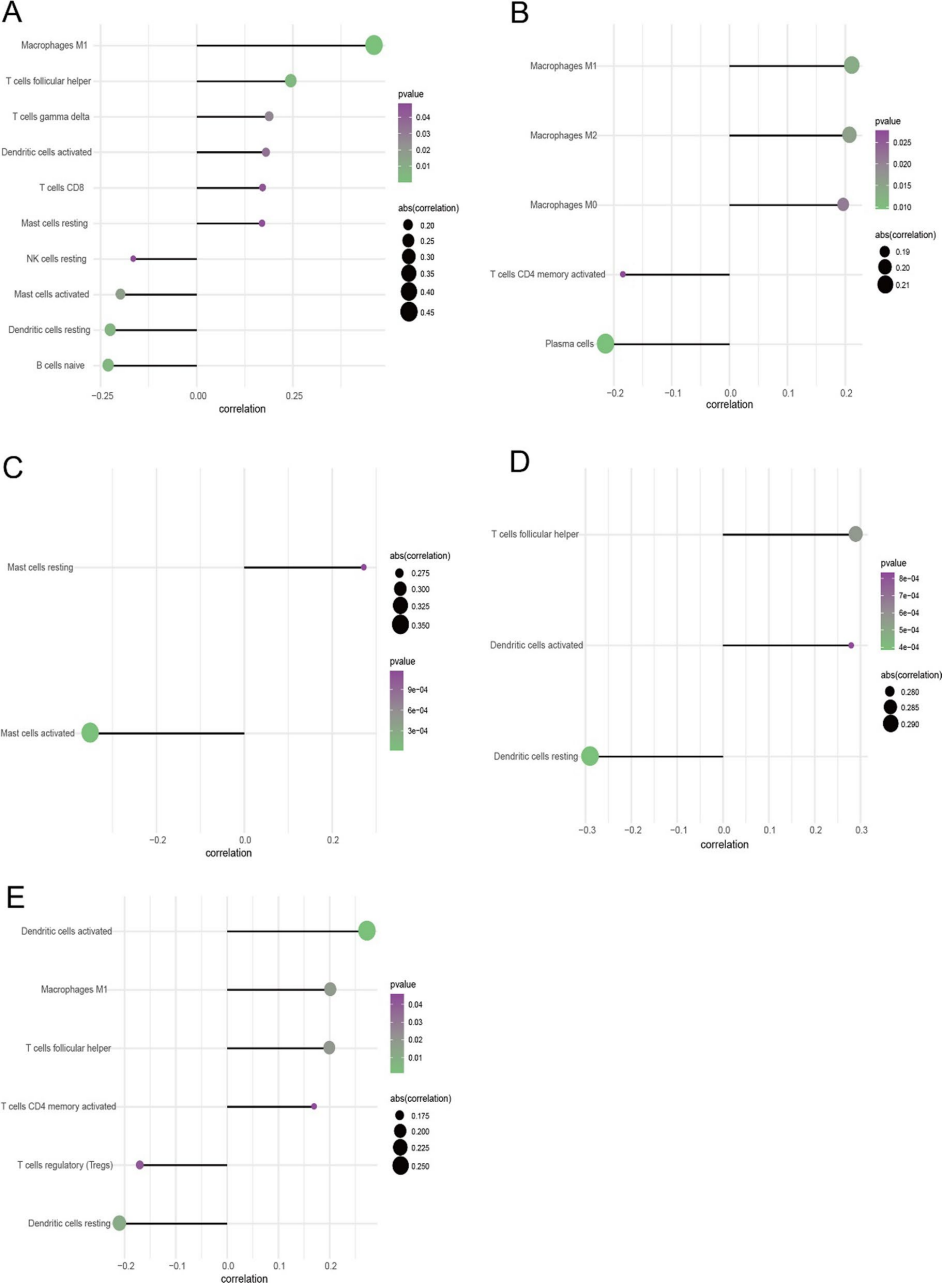
Correlation between radiosensitive key genes and immune cell content in rectal cancer. **A** Correlation of APOL6 with immune cell content. **B** Correlation of HOXC6 with immune cell content. **C** Correlation between JOSD1 and immune cell content. **D** Correlation of MATR3 with immune cell content. **E** Correlation of TOP2A with immune cell content

The tumor microenvironment is mainly composed of tumor-associated fibroblasts, immune cells, extracellular matrix, a variety of growth factors, inflammatory factors and special physical and chemical characteristics. The tumor microenvironment significantly affects the survival outcome and clinical treatment sensitivity of tumors. By analyzing the relationship between the expression level of key genes and tumor immune infiltration, the potential molecular mechanism by which the expression level of key genes affects the progression of rectal cancer was further explored. The proportion of immune cell content in each patient and the p earson correlation between immune cells are shown (Fig. 4A, B). T cells CD4 activated memory, Macrophages M0 and Macrophages M1 were significantly higher in READ patients than in normal patients, while Macrophages M2, Dendritic resting cells and Mast cells were significantly lower in READ patients than in normal patients (Fig. 4C). We further analyzed the correlation between key genes and immune cell content, and the results showed that APOL6, MATR3, and TOP2A were significantly positively correlated with T cells follicular helper and Dendritic cells activated, and significantly negatively correlated with APOL6, MATR3, and TOP2A and Dendritic cells; APOL6, HOXC6, and TOP2A were significantly positively correlated with Macrophages M1, JOSD1 and APOL6 were significantly negatively correlated with Mast cells activated (Fig. 5A–E).

### Relationship between critical genes and immunomodulatory genes

**Fig. 6:**
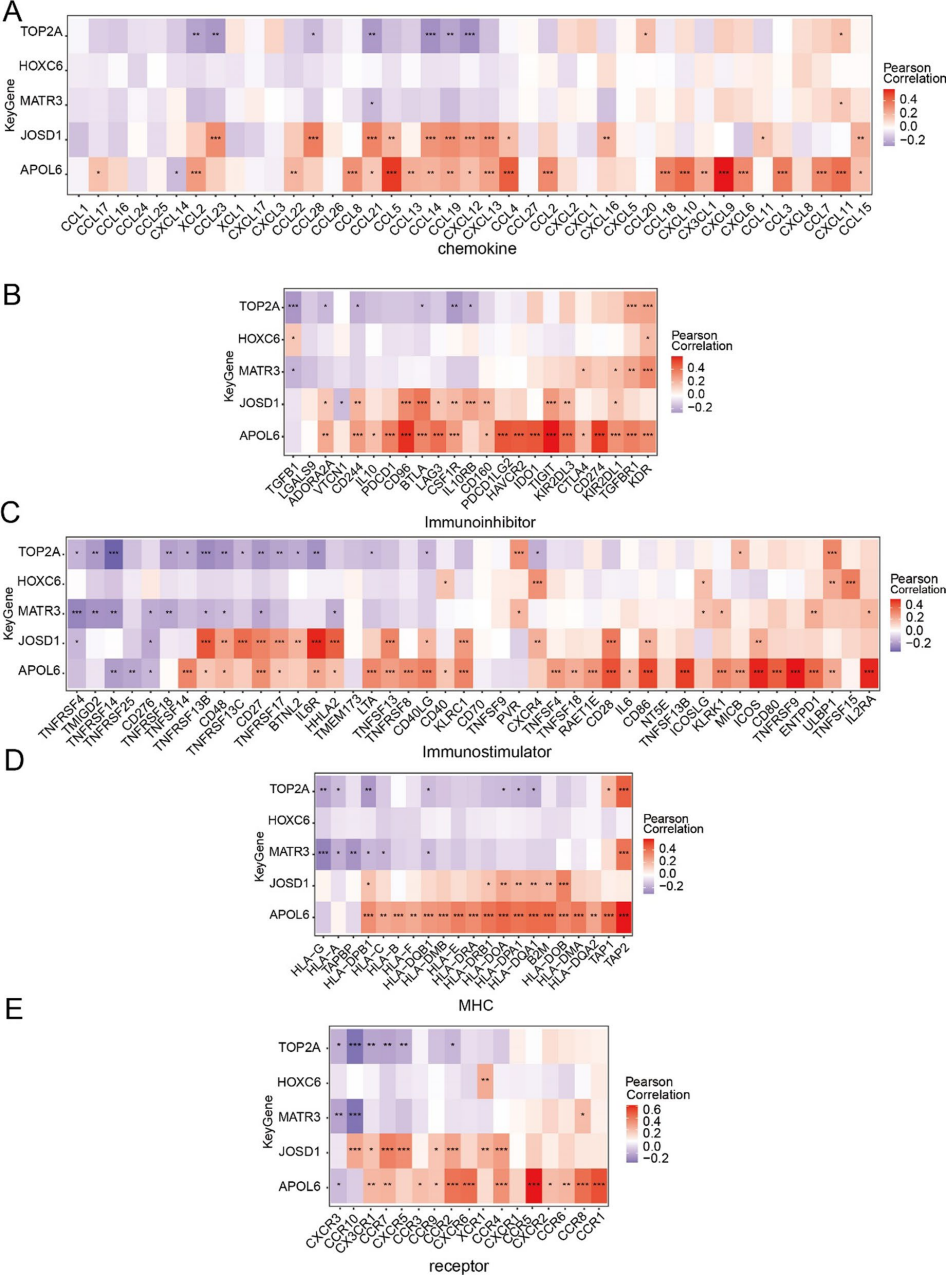
Relationship between key radiosensitive genes and immunomodulatory genes in rectal cancer. **A** Association of core genes with chemokines. **B** Correlation of core genes with immunosuppressive agents. **C** Association of core genes with immune agonists. **D** Association of core genes with MHC.**E** Association of core genes with MHC receptors

We first obtain immune factor sets for these different classes, including immune modulators, chemokines, and cellular receptors through the TISIDB database. Immunomodulatory genes were found to be highly correlated with the expression levels of key genes by correlation analysis, such as TOP2A significantly negatively correlated with chemokines, immunosuppressive agents, MHC, and MHC receptors, HOXC6 significantly positively correlated with immune activators, MATR3 significantly negatively correlated with immunosuppressive agents and MHC, and JOSD1 and APOL6 significantly positively correlated with chemokines, immunosuppressive agents, MHC, and MHC receptors (Fig. 6A–E). These analyses confirmed that key genes are closely related to the level of immune cell infiltration and play an important role in the immune microenvironment.

### Relationship between key genes and drug sensitivity

**Fig. 7:**
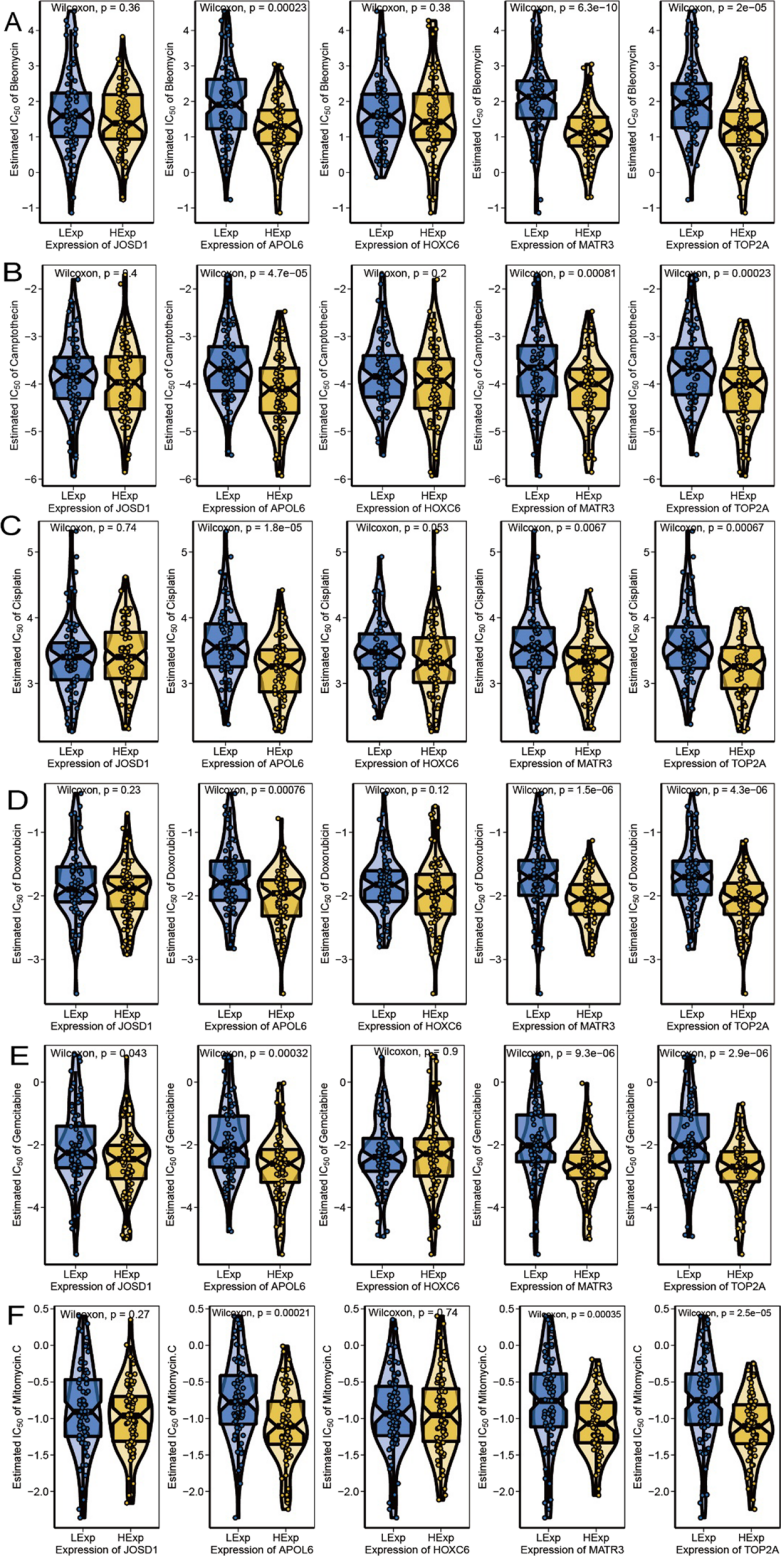
Relationship between key genes of radiosensitivity and drug sensitivity in rectal cancer. **A** Association of core genes with Bleomycin chemosensitivity. **B** Association of core genes with Camptothecin chemosensitivity. **C** Correlation of core genes with Cisplatin chemosensitivity. **D** Association of core genes with Doxorubicin chemosensitivity. **E** Association of core genes with Gemcitabine chemosensitivity. **F** Correlation of core genes with Mitomycin.C chemosensitivity

**Fig. 8:**
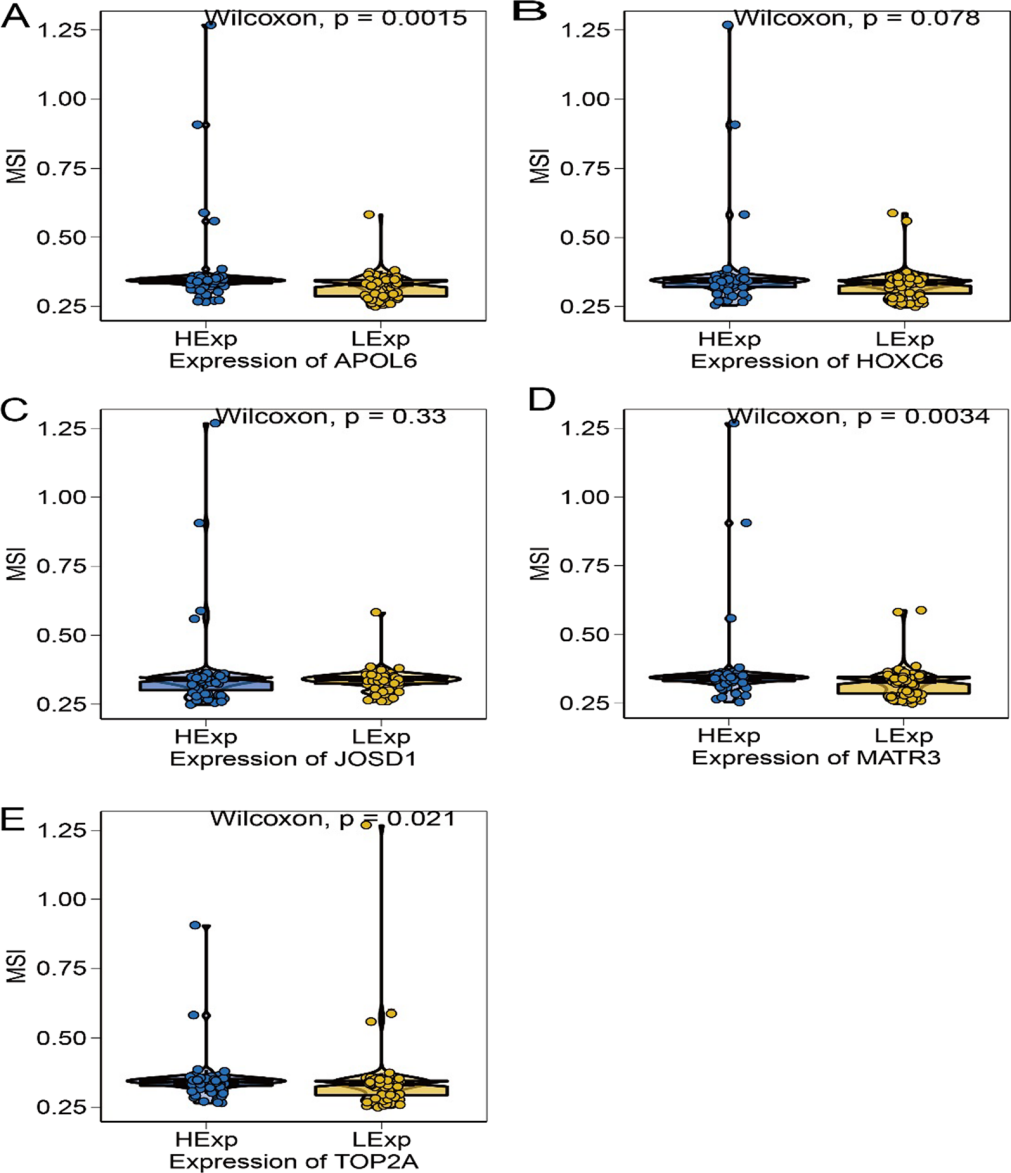
Relationship between key radiosensitive genes and microsatellite instability (MSI) in rectal cancer. **A** Association of APOL6 with MSI. **B** Correlation of HOXC6 with MSI. **C** Correlation of JOSD1 with MSI. **D** Correlation of MATR3 with MSI. **E** Correlation of TOP2A with MSI

The effect of rectal cancer surgery combined with chemotherapy is clear, and our study is based on the drug sensitivity data from GDSC database to predict the chemosensitivity of each tumor sample through R package “pRRophetic” [15], to further investigate the expression of key genes and the sensitivity of common chemotherapeutic agents. The results showed that the expression of key genes affected the sensitivity of patients to Bleomycin, Camptothecin, Cisplatin, Doxorubicin, Gemcitabine, Mitomycin.C (Fig. 7A–F). We also found significant differences in microsatellite instability (MSI) between high and low expression groups of APOL6, MATR3 and TOP2A (Fig. 8A–E).

### Discussion on specific signaling mechanisms related to key genes

**Fig. 9:**
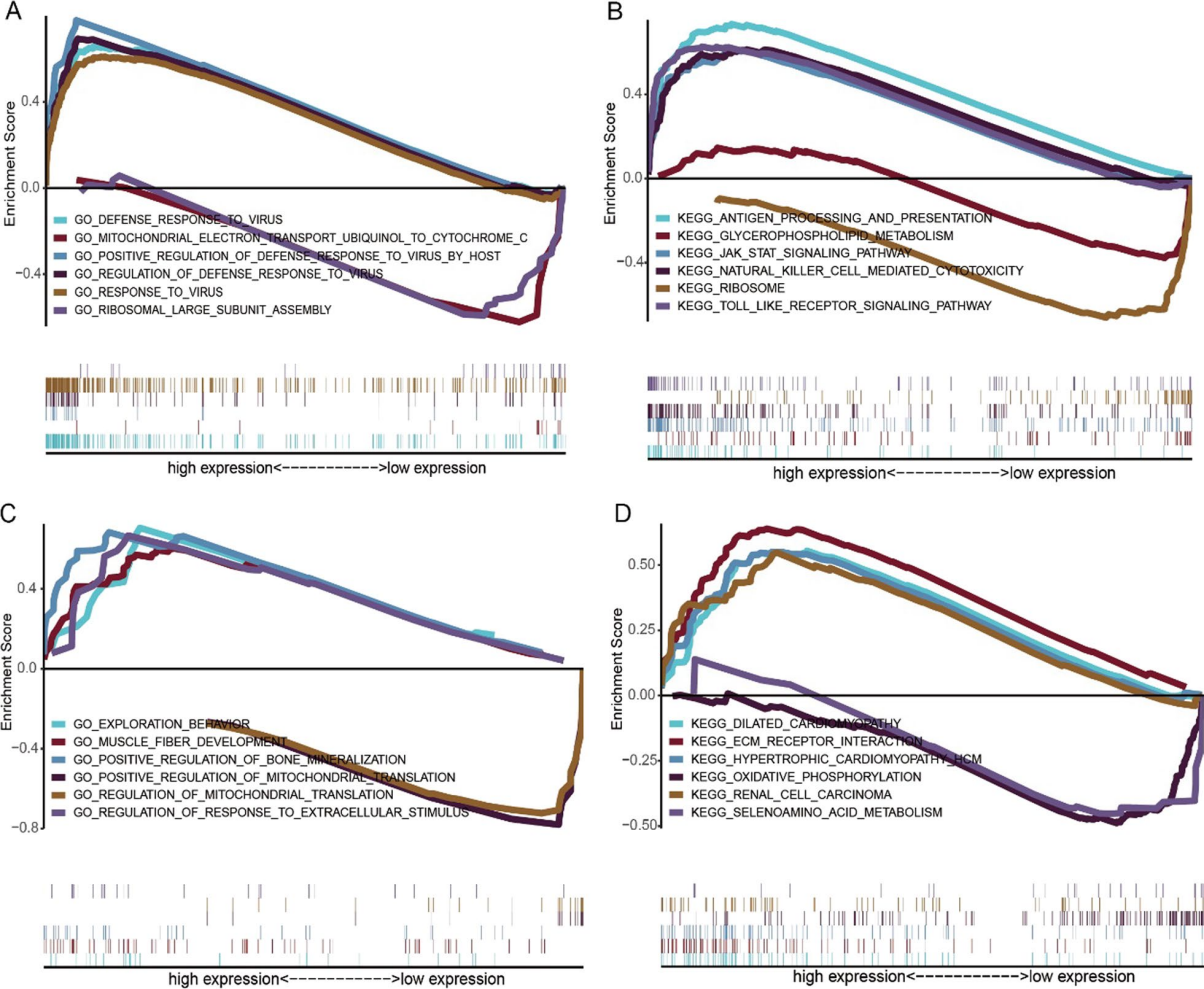
Study on specific signaling mechanism of key genes related to radiosensitivity in rectal cancer (APOL6 and HOXC6). **A** GO enriched pathway of APOL6 gene. **B** KEGG enriched pathway of APOL6 gene. **C** GO enriched pathway of HOXC6 gene. **D** KEGG enriched pathway of HOXC6 gene

**Fig. 10:**
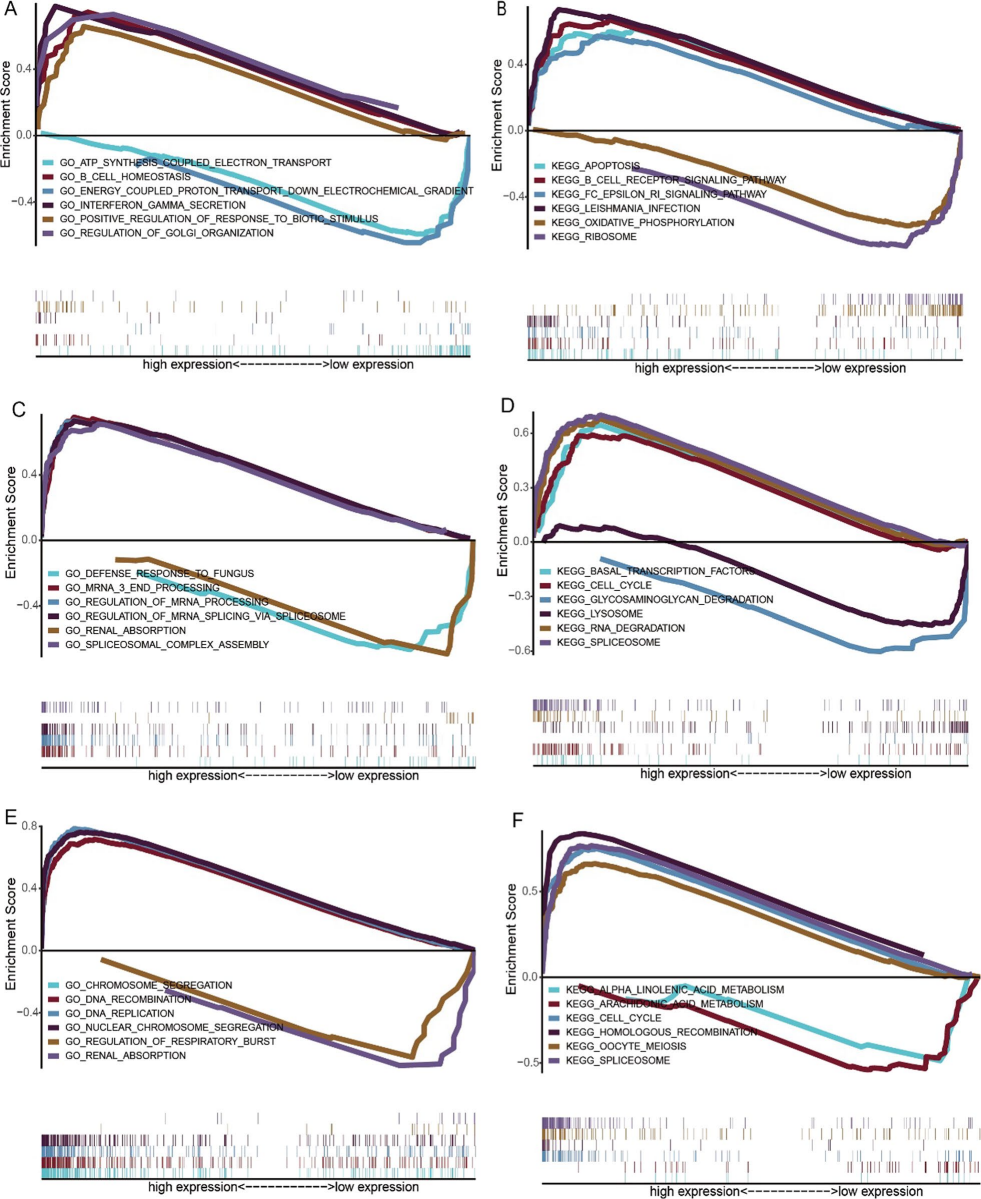
Study on specific signaling mechanism of key genes related to radiosensitivity in rectal cancer (JOSD1, MATR3 and TOP2A). **A** GO enriched pathway of JOSD1 gene. **B** KEGG enriched pathways for JOSD1 gene. **C** GO enriched pathway of MATR3 gene. **D** KEGG enriched pathway of MATR3 gene. **E** TOP2A gene GO enriched pathway. **F** TOP2A gene KEGG enriched pathway

We next explored the impact of candidate genes on disease progression-related signaling pathways for the specific signaling pathways involved in these five critical genes. The results showed that the pathways enriched by GO in APOL6 gene were defense response PRESESE virus, response to virus and other pathways (Fig. 9A), KEGG enriched pathways were antigen PRESESSING and PATNTATION, glycerophospholipid metabolism and other pathways (Fig. 9B); GO enriched pathways of HOXC6 gene include exploration behavior, muscle fiber development and other pathways (Fig. 9C), while KEGG enriched pathways include ecm receptor interaction, dilated cardiomyopathy and other pathways(Fig. 9D). GO enriched pathways of JOSD1 gene were atp synthesis coupled electron transport, b cell homeostasis and other pathways, KEGG enriched pathways were apoptosis, b cell receptor signaling pathway and other pathways; GO enriched pathways of MATR3 gene include defense response to fungus, mrna 3 end processing and other pathways, while KEGG enriched pathways include basal transacting factors, cell cycle and other pathways; The pathways enriched by GO of TOP2A gene were chromosome segregation, dna recombination and other pathways, and the pathways enriched by KEGG were alpha linolenic acid metabolism, arachidonic acid metabolism and other pathways (Fig. 10A–F).

### Construction of the critical gene-associated nomogram prediction model and clinical relevance analysis

**Fig. 11:**
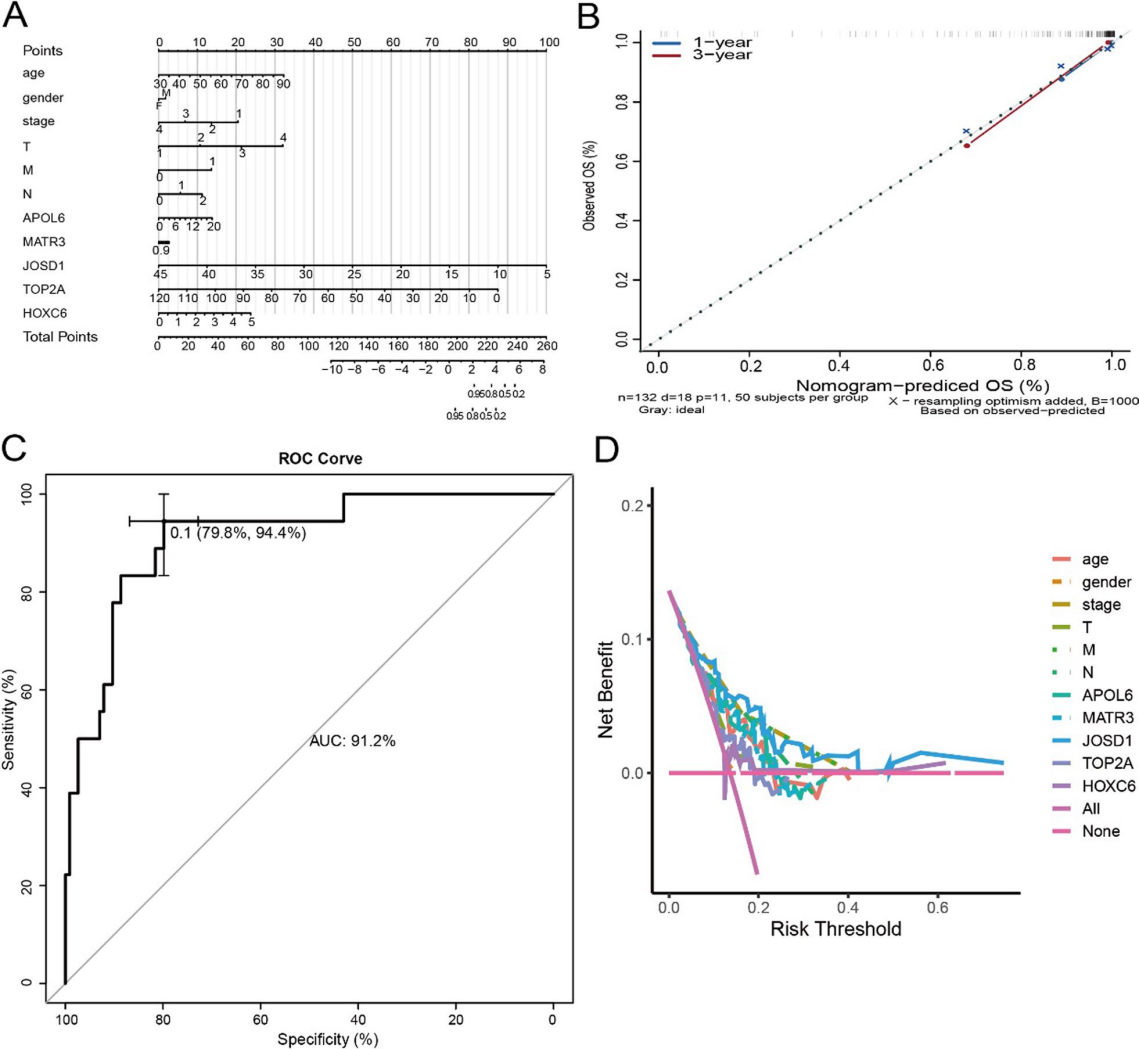
Nomogram for prediction of the outcome of patients with rectal cancer. **A** Nomogram was constructed based on the expression of APOL6, MATR3, JOSD1,TOP2A,HOXC6 and the clinical parameters. **B** Calibration curves of nomogram for predicting OS at 1-year and 3-year in the TCGA rectal cancer dataset. **C** ROC curves of the nomogram prediction model. **D** Decision curve analysis for nomogram

**Fig. 12:**
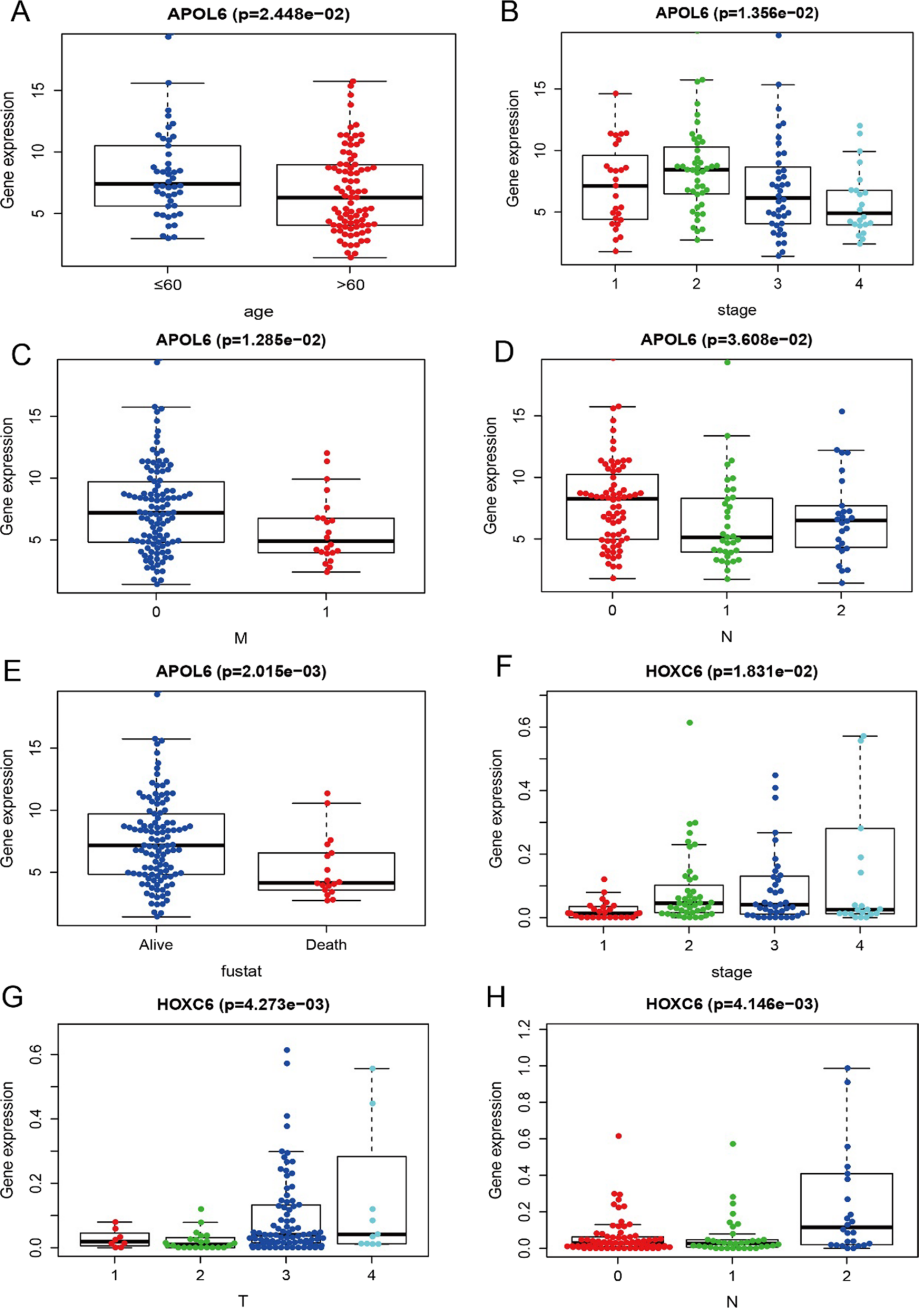
Relationship between key genes of radiosensitivity in rectal cancer and clinical indicators(APOL6 and HOXC6). **A** Association of APOL6 gene expression with age. **B** Correlation between APOL6 gene expression and tumor stage. **C** Correlation between APOL6 gene expression and M stage. **D** Correlation between APOL6 gene expression and N stage. **E** Association of APOL6 gene expression with survival status. **F** Correlation between HOXC6 gene expression and tumor stage. **G** Correlation between HOXC6 gene expression and T stage. **H** Correlation between HOXC6 gene expression and N stage

**Fig. 13:**
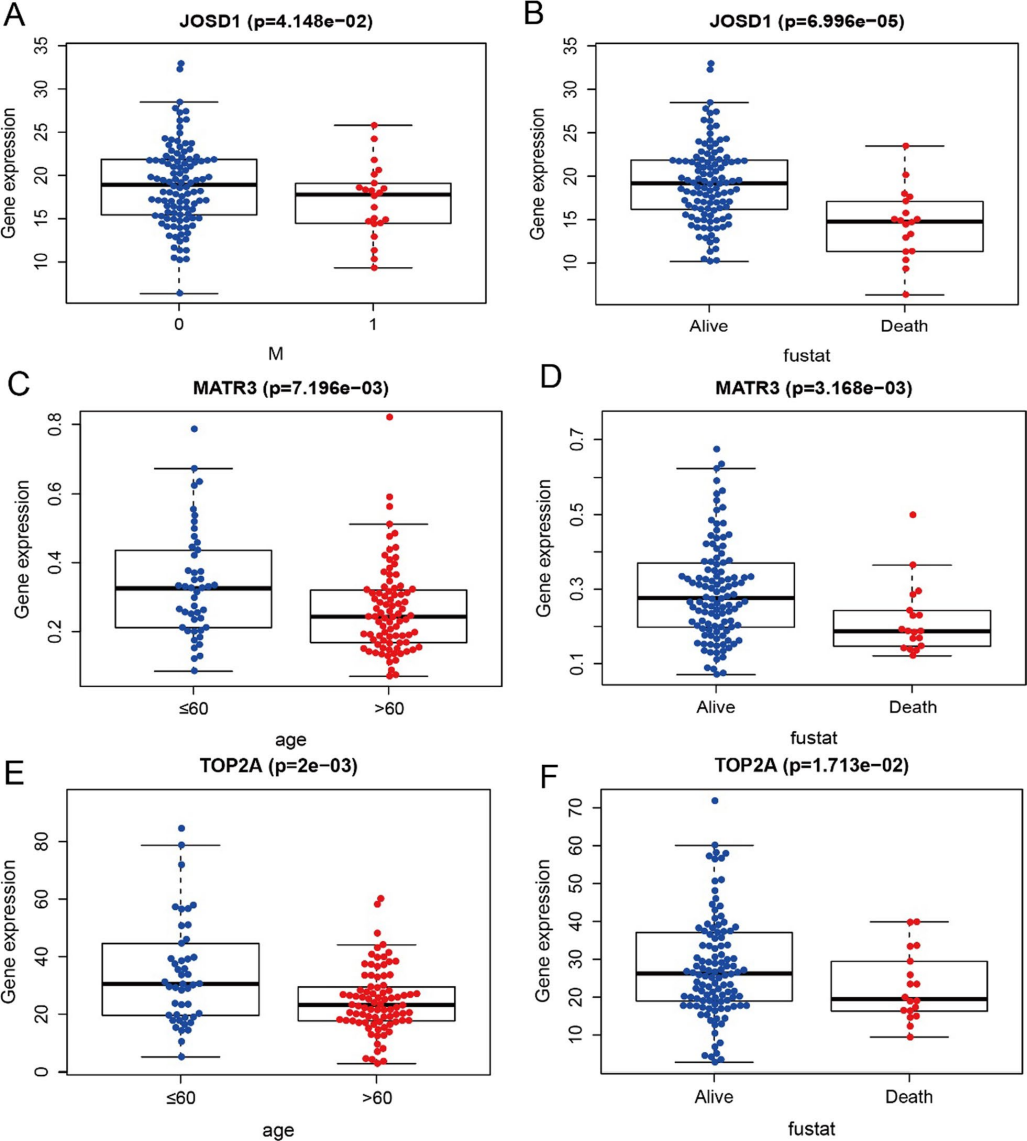
Relationship between key genes of radiosensitivity in rectal cancer and clinical indicators (JOSD1, MATR3 and TOP2A). **A** Correlation between JOSD1 gene expression and M stage. **B** Correlation between JOSD1 gene expression and survival status. **C** Correlation between MATR3 gene expression and age. **D** Correlation between MATR3 gene expression and survival status. **E** Correlation of TOP2A gene expression with age. **F** Correlation between TOP2A gene expression and survival status

In this study, nomogram model was further constructed to predict the prognosis of patients, and the results of Cox regression analysis showed that different staging patterns of rectal cancer and the expression of key genes contributed to different extents throughout the scoring process in all our samples (Fig. 11A), and predictive analysis was performed for two periods: 1 year and three years (Fig. 11B), the AUC(area under the receiver operating characteristic curve) of the nomogram model validation receiver operating characteristic (ROC) curve is 0.912 (Fig. 11C), the decision curve analysis (DCA) curves for the nomogram are presented in Fig. 11D, the DCA curve demonstrated that the nomogram had good net benefits for clinical use. In addition, we further investigated the relationship between key genes and clinical symptoms and found that the expression levels of these key genes were significant correlation between groups in multiple clinical parameters, with APOL6 significantly correlated with age, stage, M stage, N stage, and survival status of patients, HOXC6 significantly correlated with stage, T stage, and N stage of patients (Fig. 12A–H). JOSD1 significantly correlated with M stage and survival status of patients, and MATR3 and TOP2A significantly correlated with age and survival status of patients (Fig. 13A–F).

### Critical gene-associated transcriptional regulatory network analysis

**Fig. 14:**
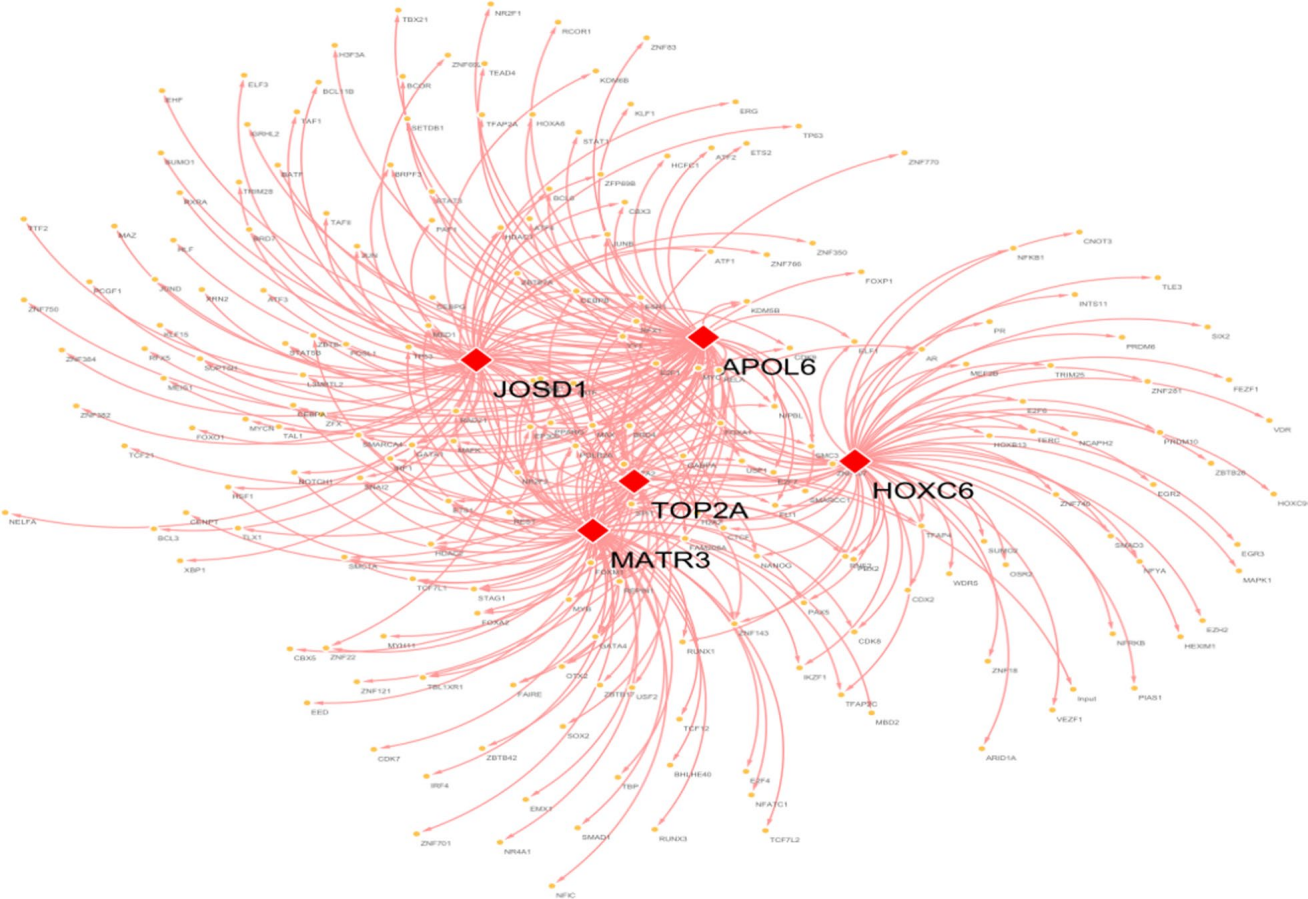
Transcriptional regulatory network of 5 core genes

We used 5 core genes as the gene set for this analysis to further investigate the transcriptional regulatory networks involved in these five core genes. Associated transcription factors were predicted by the Cistrome DB online database, with 83 transcription factors predicted by APOL6, 63 transcription factors predicted by HOXC6, 65 transcription factors predicted by JOSD1, 76 transcription factors predicted by MATR3, and 4 0 transcription factors predicted by TOP2A. Visualization by cytoscape thus constructs a comprehensive transcriptional regulatory network of key genes involved in radiotherapy in rectal cancer (Fig. 14).

### Results of qRT-PCR for key genes related to radiotherapy in rectal cancer tissues and adjacent non-cancerous tissues

**Fig. 15:**
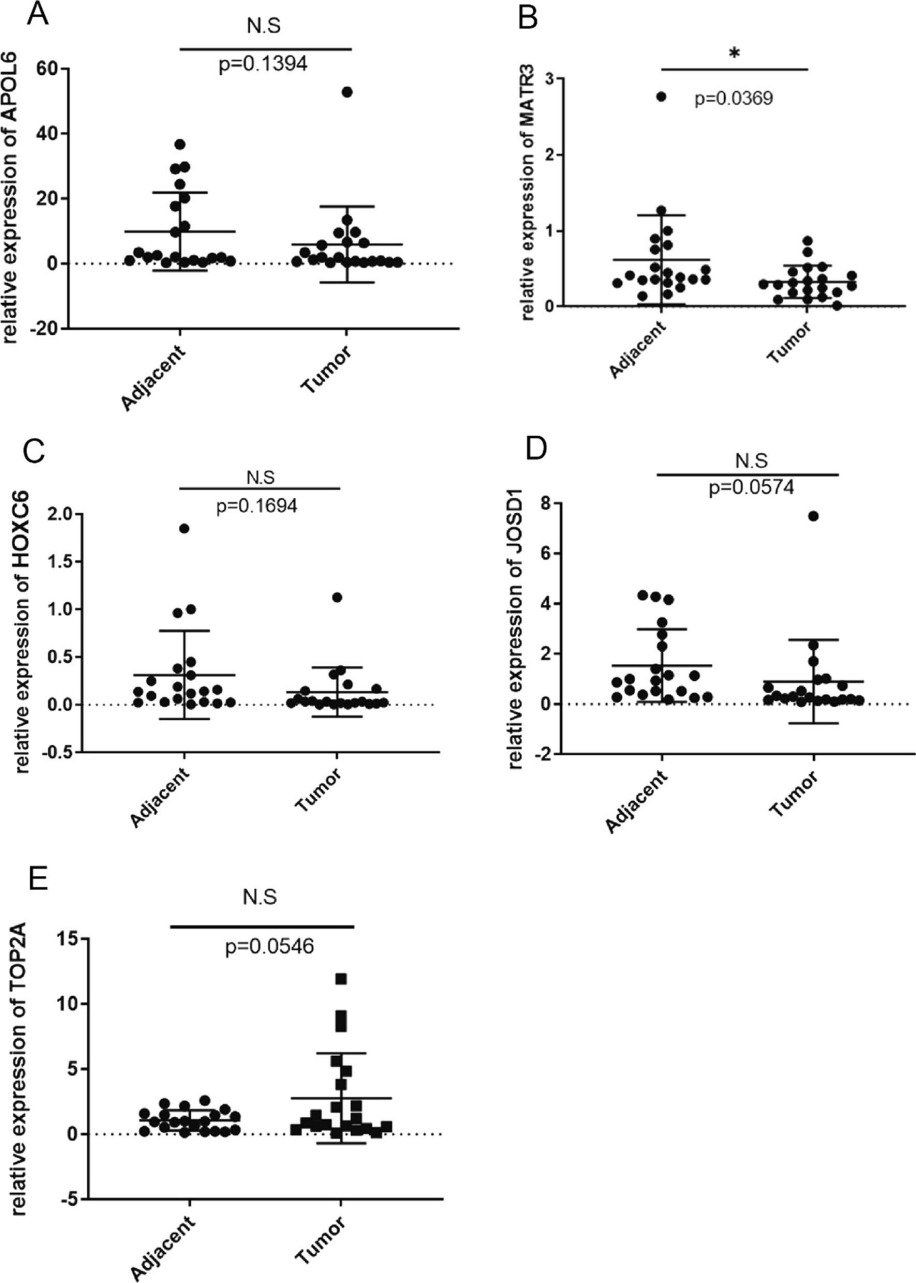
Results of qRT-PCR detection of key radiosensitive genes in rectal cancer tissues. **A** Relative expression difference of APOL6 in cancer and adjacent non-cancerous tissues. **B** Relative expression difference of MATR3 in cancer and adjacent non-cancerous tissues. **C** Relative expression difference of HOXC6 in cancer and adjacent non-cancerous tissues. **D** Relative expression difference of JOSD1 in cancer and adjacent non-cancerous tissues. **E** Relative expression difference of TOP2A in cancer and adjacent non-cancerous tissues

QRT-PCR of 5 core genes in 20 pairs of rectal adenocarcinoma tissues and adjacent non-cancerous tissues revealed that MATR3 expression was different in rectal adenocarcinoma tissues and adjacent non-cancerous tissues, with a *p* value of 0.0369, while APOL6, HOXC6, JOSD1, and TOP2A expression was not different in rectal adenocarcinoma tissues and adjacent non-cancerous tissues, with *P* values of 0.1394, 0.1694, 0.0574, and 0.0546, respectively (Fig. 15A–E).

## Discussion

In this study, we found that 600 up-regulated genes and 553 down-regulated genes were significantly different in the expression levels between the radiotherapy sensitive and radiotherapy insensitive groups of rectal cancer. Functional enrichment analysis showed that differential genes were significantly enriched in a variety of tumor biological processes. Through the random survival forest analysis of these 1153 differential genes, we finally screened five key genes, which were the radiosensitivity up-regulated genes of rectal cancer: TOP2A, MATR3, APOL6, JOSD1 and the radiosensitivity down regulated gene of rectal cancer: HOXC6.

Nuclear DNA topoisomerase II-alpha (TOP2A) is located at 17q21.2 and regulates chromosome condensation and chromatid segregation by altering the topological state of DNA during DNA replication and transcription [16]. TOP2A was found to be highly expressed in non-small cell lung cancer, hepatocellular carcinoma, and breast cancer and is associated with tumor proliferation and poor prognosis [17–19]. TOP2A is considered a major target of the chemotherapeutic agent etoposide [20]. It has also been shown that TOP2A is associated with the efficacy of radiotherapy, and Terry et al. found that TOP2A is indirectly involved in the formation of chromatid breaks in radiation-induced DNA double-strand breaks and plays a role in individual differences in chromatid radiosensitivity [21]. Upregulated expression of TOP2A is associated with recurrence after radiotherapy in prostate cancer patients [22]. Wnt/b-Zhang et al. found that inhibition of TOP2A inhibited the activity of the catenin signaling pathway in medulloblastomas, thereby reducing tumorigenicity and radioresistance of medulloblastomas [23] cells. In this study, TOP2A was found to be significantly positively associated with immune cells T cells resting helper, Dendritic follicular cells activated, Macrophages M1, and negatively associated with Dendritic follicular cells. TOP2A was significantly negatively correlated with immunomodulatory genes such as chemokines, immunosuppressive agents, MHC, MHC receptors. Correlation studies with chemosensitivity showed that TOP2A was significantly associated with chemosensitivity to bleomycin, camptothecin, cisplatin, adriamycin, gemcitabine, and mitomycin. TOP2A expression correlates with MSI. Related signal pathway enrichment analysis showed that the pathways enriched by GO of TOP2A gene included chromosome segregation and dna recombination, and the pathways enriched by KEGG included alpha linolenic acid metabolism and arachidonic acid metabolism.

Matrin3 (MATR3) is one of the nuclear matrix proteins and is a DNA and RNA-binding protein. Yang et al. found that MATR3 acts as a tumor suppressor in breast cancer cells, overexpression of MATR3 promotes apoptosis and inhibits epithelial-mesenchymal transition, migration and invasion of cells, and low expression of MATR3 is associated with poor prognosis in breast cancer patients [24]. Kuriyama et al. found that knockdown of MATR3 expression inhibited the proliferation of malignant melanoma cells [25]. Nho et al. found that decreased expression of MATR3 in oral squamous cell carcinoma cells induced apoptosis [26]. Durılewicz et al. found that low expression of MATR3 was an independent poor prognostic factor in non-small cell lung cancer [27]. In this study, MATR3 was found to be significantly positively associated with immune cells T cells follicular helper and Dendritic cells activated, and negatively associated with Dendritic cells resting. MATR3 was significantly negatively correlated with immunomodulatory genes such as immunosuppressive agents, MHC. Correlation studies with chemosensitivity identified MATR3 as significantly associated with chemosensitivity to bleomycin, camptothecin, cisplatin, doxorubicin, gemcitabine, mitomycin. MATR3 expression correlates with MSI. Related signaling pathway enrichment analysis revealed that the pathways enriched by GO of MATR3 gene were defense response funto gus, mrna 3 end processing and other pathways, and the pathways enriched by KEGG were basal transcription factors, cell cycle and other pathways.

HOXC6 is one of the members of the HOX family and maps on 12q13.3 of the human chromosome [28]. HOXC6 is aberrantly expressed in head and neck squamous cell carcinoma, gastrointestinal malignancies, and breast cancer [29, 30]. Ramachandran et al. found that low expression of HOXC6 induced apoptosis in prostate cancer cells and HOXC6 could serve as a therapeutic target for prostate cancer [31]. Several previous studies have shown that high expression of HOXC6 is associated with poor prognosis in cancer patients. Du et al. found that increased HOXC6 expression in esophageal squamous cell carcinoma predicted poor prognosis [32]. Zhang et al. found that gastric cancer patients with high HOXC6 expression had shorter survival time than patients with low HOXC6 expression [33]. Zhou et al. found that patients with high HOXC6 expression faced a higher risk of death than those with low HOXC6 expression in prostate cancer patients [34]. In this study, there was a significant positive correlation between HOXC6 and immune cells and Macrophages M1. HOXC6 was significantly positively associated with immunomodulatory genes such as immune activators. Correlation studies with chemosensitivity found no apparent correlation between HOXC6 and chemosensitivity to bleomycin, camptothecin, cisplatin, adriamycin, gemcitabine, mitomycin. HOXC6 expression did not correlate with MSI. Related signaling pathway enrichment analysis revealed that the pathways enriched by GO of HOXC6 gene were exploration behavior, muscle fiber development and ecm receptor interaction, dilated cardiomyopathy and other pathways.

APOL6 is a member of the apolipoprotein L gene family and acts as a regulator of lipid metabolism, which promotes differentiation and lipogenesis in 3T3-L1 preadipocytes [35]. Liu et al. found that the expression of APOL6 was associated with multiple programmed cell death through bioinformatics studies, suggesting that it may regulate multiple programmed cell death processes. In vitro experiments found that up-regulation of APOL6 could promote the necrosis and pyroptosis of pancreatic cancer cells, while necrosis and pyroptosis could enhance anti-tumor immune effects, which also indirectly explained the up-regulation of APOL6 in immunotherapy responders [36]. Grace R. Raji et al. found that miR-643 is horizontally transferred from cisplatin-resistant cells and confers chemoresistance in receptor drug-sensitive cells by targeting APOL6 [37]. In this study, significant positive correlations were found between APOL6 and immune cells T cells follicular helper, Dendritic cells activated, Macrophages M1, and significant negative correlations with Dendritic resting cells, Mast activated cells, etc. APOL6 was significantly positively associated with immunomodulatory genes such as chemokines, immunosuppressive agents, MHC, MHC receptors. Correlation studies with chemosensitivity identified APOL6 as significantly associated with chemosensitivity to bleomycin, camptothecin, cisplatin, adriamycin, gemcitabine, mitomycin. APOL6 expression correlates with MSI. Related signaling pathway enrichment analysis revealed that the pathways enriched by GO of APOL6 gene were defense response to virus, response to virus and other pathways, and the pathways enriched by KEGG were antigen processing and presentation, glycerophospholipid metabolism and other pathways.

JOSD1 is a member of the smallest family of DUBs MJDs, which contains only a highly conserved Josephine domain located 22 q13.1 on human chromosome [38, 39]. Mutations in JOSD1 gene have been identified in melanoma, endometrial, bladder, and ovarian cancers [38]. Previous studies have shown that JOSD1 stabilizes target proteins by cleaving the K48 ubiquitin chain [40, 41]. JOSD1 promotes chemoresistance by stabilizing MCL1 in gynecologic tumors [40]. Jing et al. found that under epigenetic regulation of BRD4, JOSD1 was overexpressed in HNSCC, and increased expression of JOSD1 was positively correlated with proliferation and chemoresistance of HNSCC cells, while highly expressed JOSD1 was also associated with poor prognosis of HNSCC patients [42]. In this study, JOSD1 was found to be negatively correlated with Mast cells activated significantly. JOSD1 was significantly positively associated with immunomodulatory genes such as chemokines, immunosuppressive agents, MHC, MHC receptors. Correlation studies with chemosensitivity revealed that JOSD1 was significantly associated with chemosensitivity to gemcitabine, but not to bleomycin, camptothecin, cisplatin, adriamycin, or mitomycin. Expression of JOSD1 did not correlate with microsatellite instability (MSI). Related signaling pathway enrichment analysis revealed that: the pathways enriched in GO of JOSD1 gene were atp synthesis coupled electron transport, b cell homeostasis and pathways enriched in KEGG were apoptosis, b cell receptor signaling pathway.

We investigated the association of five core genes with clinical parameters and found that APOL6 was significantly associated with patient age, overall stage, M stage, N stage, and survival status, HOXC6 was significantly associated with patient overall stage, T stage, and N stage, JOSD1 was significantly associated with patient M stage and survival status, and MATR3 and TOP2A were significantly associated with patient age as well as survival status. At the same time, we constructed nomogram model to predict the prognosis of patients, and the age, gender, total stage of rectal cancer, T stage, N stage, M stage and the expression of five key genes of patients contributed to different extents, and predicted the 1- and 3-year survival, indicating that nomogram has a good predictive efficacy and can guide clinical practice. Patients with microsatellite instability-high (MSI-H) have a high tumor mutation burden and increased numbers of tumor-infiltrating lymphocytes and exhibit high sensitivity to immunotherapy [43, 44]. The keynote-177 study has shown that pembrolizumab has emerged as a new standard of first-line treatment for patients with metastatic dMMR/MSI-H colorectal cancer [45]. We found that TOP2A, APOL6, MATR3 were correlated with MSI and could be used as potential indicators of sensitivity to immunotherapy for rectal cancer. In parallel, we visualized cytoscape to construct a comprehensive transcriptional regulatory network of five key genes involved in radiotherapy in rectal cancer.

We found a new key gene related to the prognosis of rectal cancer, which may be a new biomarker for rectal cancer. We validated the expression of five core genes in human rectal cancer tissue samples in cancer and adjacent non-cancerous tissues and found that MATR3 expression was significant difference and there was no significant difference in the expression of the remaining four core genes. However, our study still has some limitations. First, the sample size retrieved from the database was limited. Second, the findings lack in vitro and in vivo experimental validation. In subsequent studies, we will perform cell function experiments, xenograft experiments in nude mice, and molecular mechanism experiments on irradiated human rectal cancer cell lines after overexpression or knockdown of TOP2A, MATR3, APOL6, JOSD1, and HOXC6. Despite these shortcomings, preliminary findings can still provide very meaningful and constructive information.

## Conclusion

In summary, we identified five radiosensitivity-related genes associated with rectal cancer prognosis: TOP2A, MATR3, APOL6, JOSD1, HOXC6. The relationship of core genes with immune infiltration, immune-related genes, chemotherapeutic drug sensitivity, enriched signaling pathways and transcriptional regulatory networks is systematically clarified. In addition, we constructed prognostic nomograms of core genes versus clinicopathologic features, established calibration curves, and predicted survival in rectal cancer patients. At the same time, we preliminarily verified the expression of core genes in rectal cancer tissues and adjacent non-cancerous tissues. We have a preliminary, but relatively comprehensive understanding of genes involved in radiosensitivity in rectal cancer. Next, we will further investigate the molecular mechanism of rectal radiosensitivity-related genes through in vitro cell experiments and animal experiments.

## Data Availability

The datasets used and/or analysed during the current study available from the corresponding author on reasonable request.
